# K_0.8_Ag_0.2_Nb_4_O_9_AsO_4_

**DOI:** 10.1107/S1600536808015225

**Published:** 2008-05-24

**Authors:** Rym Ben Amor, Mohamed Faouzi Zid, Ahmed Driss

**Affiliations:** a Laboratoire de Matériaux et Cristallochimie, Faculté des Sciences, Université de Tunis – El-Manar, 2092 El-Manar, Tunis, Tunisie

## Abstract

The title compound, potassium silver tetra­niobium nona­oxide arsenate, K_0.8_Ag_0.2_Nb_4_O_9_AsO_4_, was prepared by a solid-state reaction at 1183 K. The structure consists of infinite (Nb_2_AsO_14_)_*n*_ chains parallel to the *b* axis and cross-linked by corner sharing *via* pairs of edge-sharing octa­hedra. Each pair links together four infinite chains to form a three-dimensional framework. The K^+^ and Ag^+^ ions partially occupy several independent close positions in the inter­connected cavities delimited by the framework. K_0.8_Ag_0.2_Nb_4_O_9_AsO_4_ is likely to exhibit fast alkali-ion mobility and ion-exchange properties. The Wyckoff symbols of special positions are as follows: one Nb 8*e*, one Nb 8*g*, As 4*c*, two K 8*f*, one Ag 8*f*, one Ag 4*c*, one O 8*g*, one O 4*c*.

## Littérature associée

Pour le contexte général du travail et structures associées, voir: Ben Amor & Zid (2006 ▶); Benhamada *et al.* (1992 ▶); Bestaoui *et al.* (1998 ▶); Brown & Altermatt (1985 ▶); Haddad, Jouini & Ghedira (1988 ▶); Haddad, Jouini *et al.* (1988 ▶); Harrison *et al.* (1994 ▶); Ledain *et al.* (1997 ▶); Piffard *et al.* (1985 ▶); Zid *et al.* (1989 ▶, 1992 ▶, 1998 ▶, 2005 ▶).

## Partie expérimentale

### 

#### Données cristallines

K_0.8_Ag_0.2_Nb_4_O_9_AsO_4_
*M* *_r_* = 707.41
Orthorhombique, 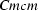

*a* = 10.469 (2) Å
*b* = 10.403 (2) Å
*c* = 10.047 (1) Å
*V* = 1094.2 (3) Å^3^
*Z* = 4
Radiation Mo *K*α
μ = 7.81 mm^−1^
*T* = 298 (2) K
0.20 × 0.14 × 0.10 mm

#### Collection des données

Diffractomètre Enraf–Nonius CAD-4
Correction d’absorption: ψ scan (North *et al.*, 1968 ▶) *T*_min_ = 0.24, *T*_max_ = 0.45
2162 réflexions mesurées
803 réflexions independantes
751 réflexions avec *I* > 2σ(*I*)
*R*_int_ = 0.024
2 réflexions de référence fréquence: 120 min variation d’intensité: 0.9%

#### Affinement

*R*[*F* ^2^ > 2σ(*F* ^2^)] = 0.024
*wR*(*F* ^2^) = 0.067
*S* = 1.13
803 réflexions
68 paramètres
Δρ_max_ = 0.90 e Å^−3^
Δρ_min_ = −1.64 e Å^−3^

### 

Collection des données: *CAD-4 EXPRESS* (Duisenberg, 1992 ▶; Macíček & Yordanov, 1992 ▶); affinement des paramètres de la maille: *CAD-4 EXPRESS*; réduction des données: *XCAD4* (Harms & Wocadlo, 1995 ▶); programme(s) pour la solution de la structure: *SHELXS97* (Sheldrick, 2008 ▶); programme(s) pour l’affinement de la structure: *SHELXL97* (Sheldrick, 2008 ▶); graphisme moléculaire: *DIAMOND* (Brandenburg, 1998 ▶); logiciel utilisé pour préparer le matériel pour publication: *WinGX* (Farrugia, 1999 ▶).

## Supplementary Material

Crystal structure: contains datablocks I, global. DOI: 10.1107/S1600536808015225/fj2116sup1.cif

Structure factors: contains datablocks I. DOI: 10.1107/S1600536808015225/fj2116Isup2.hkl

Additional supplementary materials:  crystallographic information; 3D view; checkCIF report

## Figures and Tables

**Table 1:** Paramètres géométriques (Å)

Nb1—O3^i^	1.813 (3)
Nb1—O4	2.001 (3)
Nb1—O1	2.256 (2)
Nb2—O4	1.849 (3)
Nb2—O5	1.932 (2)
Nb2—O3	2.122 (3)
Nb2—O2	2.128 (4)
As1—O2^i^	1.672 (4)
As1—O1	1.707 (4)
K1—O3^ii^	2.764 (5)
K1—O3	2.869 (6)
K1—O2^iii^	2.989 (6)
K2—O5^iv^	2.48 (3)
K2—O3^iv^	2.630 (9)
K2—O2^v^	2.70 (2)
Ag1—O5^iv^	2.272 (11)
Ag1—O2^v^	2.500 (10)
Ag2—O5^iv^	2.188 (15)
Ag2—O2^v^	2.386 (13)

Codes de symétrie : (i) 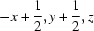; (ii) 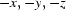; (iii) 
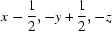
; (iv) 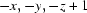; (v) 
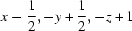
.

**Fig. 1.**
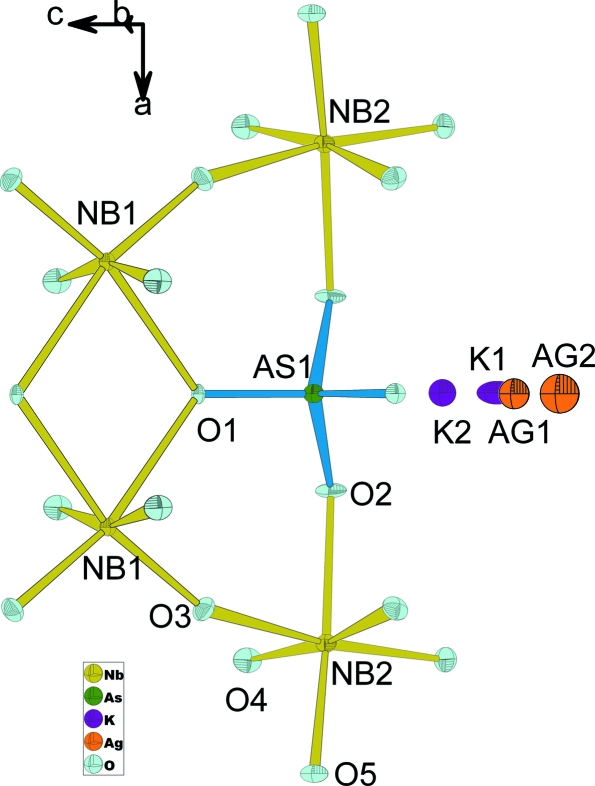
Unité asymétrique dans K0.8Ag0.2Nb4O9AsO4.

**Fig. 2.**
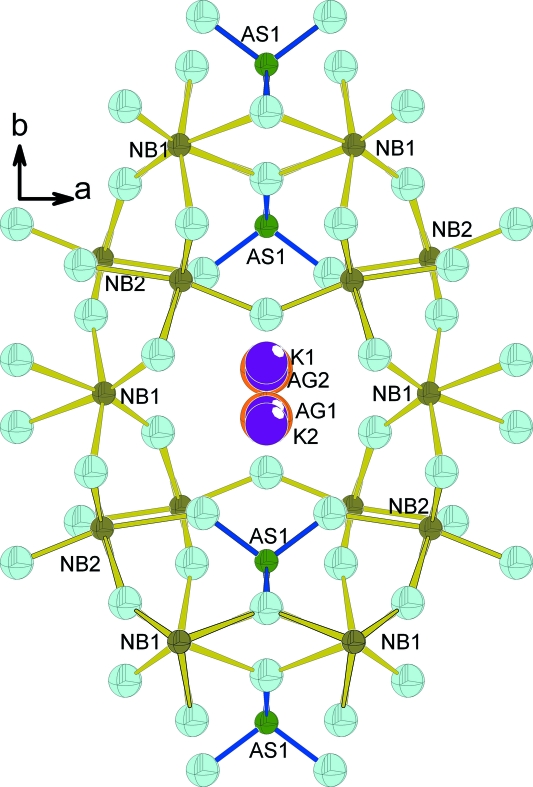
Représentation d'un cycle formé par les octaèdres NbO6 montrant les cavités élliptiques où logent les cations.

**Fig. 3.**
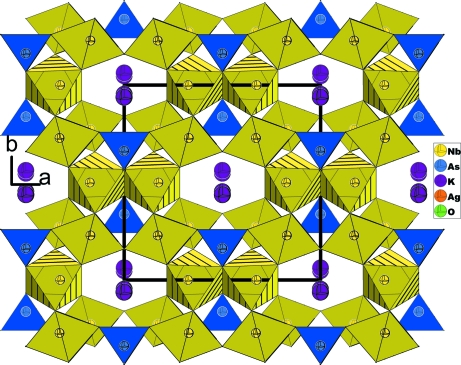
Projection de la stucture de K0.8Ag0.2Nb4O9AsO4 selon c montrant les cavités où résident les cations.

## supplementary crystallographic information

### Comment

La recherche de nouveaux matériaux à charpentes mixtes formées d'octaèdres MO_6_ (*M* = métal de transition) et de tétraèdres XO_4_ (*X* = P, As) suscite un grand intérêt ces dernières années (Benhamada *et al.*, 1992; Harrison *et al.*, 1994; Zid *et al.*, 1998). En effet, la jonction entre ces polyèdres conduit à des composés à charpentes ouvertes présentant de nombreuses propriétés physico-chimiques intéressantes qui sont en relation directe avec leurs structures cristallines notamment: conduction ionique (Piffard *et al.*, 1985), échange d'ions (Haddad, Jouini & Ghedira, 1988) et parfois comme produits d'intercalation en catalyse hétérogène (Ledain *et al.*, 1997). C'est dans ce cadre que nous avons exploré les systèmes A–Nb–As–O (A = Cation monovalent) dans lesquels nous avons précédemment caractérisé les phases suivantes: K_3_NbAs_2_O_9_ (Zid *et al.*, 1989), K_3_NbP_2_O_9_ (Zid *et al.*, 1992) et Ag_3_Nb_3_As_2_O_14_ (Ben Amor *et al.*, 2006).

A fin d'augmenter la mobilité des cations en passant à une occupation partielle des sites dans la structure nous avons choisi d'introduire avec le cation alcalin un métal monovalent de transition Ag. Dans ce travail nous nous sommes intéressés en premier à la synthèse et l'étude structurale sur monocristal du matériau puis à l'étude de l'influence de l'introduction d'un métal de transition monovalent sur ces propriétés physiques notamment de conduction ionique. Le composé K_0.8_Ag_0.2_Nb_4_O_9_AsO_4_ obtenu est de formulation et de symétrie similaires à celles de KNb_4_O_9_AsO_4_ (Haddad, Jouini *et al.*, 1988) et NaNb_4_O_9_AsO_4_ (Bestaoui *et al.*, 1998).

L'unité asymétrique du composé K_0.8_Ag_0.2_Nb_4_O_9_AsO_4_ est construite au moyen d'un tétraèdre As(1)O_4_ relié par mise en commun de sommets d'une part à deux octaèdres Nb(2)O_6_ partageant un sommet et d'autre part à un groupement Nb(1)_2_O_10_ formé à partir d'une paire d'octaèdres Nb(1)O_6_ partageant une arête (Fig. 1).

La structure du composé K_0.8_Ag_0.2_Nb_4_O_9_AsO_4_ peut être décrite au moyen de chaînes infinies (Nb(2)_2_AsO_14_)_n_ de type (Nb(2)O_6_–Nb(2)O_6_–As(1)O_4_) disposées selon la direction [100], reliées entre elles par mise en commun de sommets avec des paires d'octaèdres Nb(1)O_6_ partageant une arête. De plus, les atomes d'oxygène formant l'arête commune des paires appartiennent aussi aux tétraèdres AsO_4_. Notons qu'au sein de la charpente anionique (Nb_4_O_9_AsO_4_)^-^, les octaèdres Nb(1)O_6_ et Nb(2)O_6_ se lient par partage de sommets en developpant des cycles d'octaèdres (Fig. 2). Il en résulte ainsi une charpente tridimensionnelle possédant des canaux, à section hexagonale, communicants où résident les cations K^+^ et Ag^+^ (Fig. 3).

Le calcul des différentes valences des liaisons utilisant la formule empirique de Brown (Brown & Altermatt, 1985) vérifie bien les valeurs de charges des ions dans la phase étudiée.

La structure est iso-structurale à celle au potassium KNb_4_O_9_AsO_4_ (Haddad, Jouini *et al.*, 1988), cependant la disposition des cations et leurs coordinences sont différentes. On remarque que dans le composé KNb_4_O_9_AsO_4_ les ions occupent la même position spéciale 8f du groupe d'espace *Cmcm*, alors que dans la structure étudiée les ions occupent statistiquement plusieurs positions de part et d'autre de celle 8f ci-dessus indiquée (Fig. 3). Cette répartition des cations sur plusieurs positions pourrait conduire à une forte mobilité, par conséquent à un nouveau bon conducteur ionique (Zid *et al.*, 2005). Des mesures éléctriques moyennant un pont d'impédance complexe de type HP4192A seront réalisées dès l'obtention d'une phase pure.

### Experimental

Les cristaux relatifs à K_0.8_Ag_0.2_Nb_4_O_9_AsO_4_ ont été obtenus à partir d'un mélange formé des réactifs: Nb_2_O_5_ (Fluka, 72520), NH_4_H_2_AsO_4_ (préparé au laboratoire, ASTM 01-775), K_2_CO_3_ (Fluka, 60109) et AgNO_3_ (Merk, 101510) pris dans les rapports molaires K:Ag:Nb:As égaux à 1:1:4:1. Le mélange, finement broyé, est préchauffé à l'air à 673 K en vue d'éliminer NH_3_, H_2_O, CO_2_ et NO_2_. Il est ensuite porté jusqu'à une température de synthèse proche de la fusion, 1183 K. Le mélange est alors abandonné à cette température pendant deux semaines pour favoriser la germination des cristaux. Le résidu final a subi en premier un refroidissement lent (5°/h) jusqu'à 1173 K puis un second rapide (50°/h) jusqu'à la température ambiante. Des cristaux incolores, de taille suffisante pour les mesures des intensités, ont été séparés du flux par l'eau bouillante. Une analyse qualitative au M.E.B.E. de type FEI Quanta 200 confirme la présence des différents éléments chimiques attendus: As, Nb, K, Ag et l'oxygène.

### Refinement

L'analyse des Fouriers différences finales révèle l'existence de certains pics résiduels très proche des positions des ions K^+^. L'affinement de la structure a été donc mené lentement utilisant un par un les différents pics résiduels parus. Un affinement final a été réalisé, avec seulement quatre ions occupant statistiquement des positions proches avec un taux d'occupation globale vérifiant bien la condition de la neutralité électrique. Il conduit à un résultat très satisfaisant. Notons que dans le dernier affinement et à cause des taux d'occupation faibles des cations K(2), Ag(1) et Ag(2), nous avons choisi des agitations thermiques isotropes. Par ailleurs les ellipsoïdes sont mieux définies.

### Crystal data

**Table:**

K_0.8_Ag_0.2_Nb_4_O_9_AsO_4_	*F*_000_ = 1302
*M**_r_* = 707.41	*D*_x_ = 4.294 Mg m^−^^3^
Orthorhombic, *C**m**c**m*	Mo *K*α radiation λ = 0.71073 Å
Hall symbol: -C 2c 2	Cell parameters from 25 reflections
*a* = 10.469 (2) Å	θ = 12–16º
*b* = 10.403 (2) Å	µ = 7.81 mm^−^^1^
*c* = 10.047 (1) Å	*T* = 298 (2) K
*V* = 1094.2 (3) Å^3^	Prism, colourless
*Z* = 4	0.20 × 0.14 × 0.10 mm

### Data collection

**Table:**

Enraf–Nonius CAD-4 diffractometer	*R*_int_ = 0.024
Radiation source: fine-focus sealed tube	θ_max_ = 29.0º
Monochromator: graphite	θ_min_ = 2.8º
*T* = 298(2) K	*h* = −14→14
ω/2θ scans	*k* = −1→14
Absorption correction: ψ scan(North *et al.*, 1968)	*l* = −13→3
*T*_min_ = 0.24, *T*_max_ = 0.45	2 standard reflections
2162 measured reflections	every 120 min
803 independent reflections	intensity decay: 0.9%
751 reflections with *I* > 2σ(*I*)	

### Refinement

**Table:**

Refinement on *F*^2^	Secondary atom site location: difference Fourier map
Least-squares matrix: full	*w* = 1/[σ^2^(*F*_o_^2^) + (0.0323*P*)^2^ + 10.2211*P*] where *P* = (*F*_o_^2^ + 2*F*_c_^2^)/3
*R*[*F*^2^ > 2σ(*F*^2^)] = 0.024	(Δ/σ)_max_ < 0.001
*wR*(*F*^2^) = 0.067	Δρ_max_ = 0.90 e Å^−^^3^
*S* = 1.13	Δρ_min_ = −1.64 e Å^−^^3^
803 reflections	Extinction correction: SHELXL97 (Sheldrick, 2008), Fc^*^=kFc[1+0.001xFc^2^λ^3^/sin(2θ)]^-1/4^
68 parameters	Extinction coefficient: 0.0031 (2)
Primary atom site location: structure-invariant direct methods	

### Special details

**Table:**

Geometry. All e.s.d.'s (except the e.s.d. in the dihedral angle between two l.s. planes) are estimated using the full covariance matrix. The cell e.s.d.'s are taken into account individually in the estimation of e.s.d.'s in distances, angles and torsion angles; correlations between e.s.d.'s in cell parameters are only used when they are defined by crystal symmetry. An approximate (isotropic) treatment of cell e.s.d.'s is used for estimating e.s.d.'s involving l.s. planes.
Refinement. Refinement of *F*^2^ against ALL reflections. The weighted *R*-factor *wR* and goodness of fit *S* are based on *F*^2^, conventional *R*-factors *R* are based on *F*, with *F* set to zero for negative *F*^2^. The threshold expression of *F*^2^ > σ(*F*^2^) is used only for calculating *R*-factors(gt) *etc*. and is not relevant to the choice of reflections for refinement. *R*-factors based on *F*^2^ are statistically about twice as large as those based on *F*, and *R*-factors based on ALL data will be even larger.

### Fractional atomic coordinates and isotropic or equivalent isotropic displacement parameters (Å^2^)

**Table:**

	*x*	*y*	*z*	*U*_iso_*/*U*_eq_	Occ. (<1)
Nb1	0.17473 (4)	0.5000	0.0000	0.00668 (16)	
Nb2	0.17121 (4)	0.22837 (4)	0.2500	0.00689 (16)	
As1	0.0000	0.66228 (7)	0.2500	0.0081 (2)	
K1	0.0000	0.0628 (6)	−0.0585 (8)	0.0213 (10)	0.29
K2	0.0000	0.0484 (15)	0.871 (5)	0.024 (5)*	0.111 (15)
Ag1	0.0000	0.0492 (8)	0.682 (2)	0.021 (3)*	0.070 (6)
Ag2	0.0000	0.0513 (14)	0.7500	0.031 (5)*	0.064 (6)
O1	0.0000	0.5676 (4)	0.1112 (4)	0.0077 (7)	
O2	0.3722 (3)	0.2588 (4)	0.2500	0.0114 (8)	
O3	0.2170 (3)	0.0783 (3)	0.1151 (3)	0.0114 (5)	
O4	0.1491 (3)	0.3420 (3)	0.1103 (3)	0.0124 (6)	
O5	0.0000	0.1590 (5)	0.2500	0.0112 (11)	

### Atomic displacement parameters (Å^2^)

**Table:**

	*U*^11^	*U*^22^	*U*^33^	*U*^12^	*U*^13^	*U*^23^
Nb1	0.0062 (2)	0.0079 (2)	0.0059 (3)	0.000	0.000	−0.00167 (15)
Nb2	0.0065 (2)	0.0056 (2)	0.0085 (3)	0.00101 (15)	0.000	0.000
As1	0.0069 (3)	0.0087 (4)	0.0086 (4)	0.000	0.000	0.000
K1	0.010 (2)	0.030 (3)	0.024 (3)	0.000	0.000	0.001 (2)
O1	0.0081 (16)	0.0094 (16)	0.0057 (17)	0.000	0.000	−0.0051 (14)
O2	0.0039 (16)	0.0103 (18)	0.020 (2)	0.0024 (13)	0.000	0.000
O3	0.0111 (12)	0.0123 (12)	0.0110 (13)	0.0010 (11)	0.0026 (10)	−0.0036 (10)
O4	0.0119 (12)	0.0123 (13)	0.0130 (15)	−0.0001 (11)	−0.0003 (11)	0.0069 (11)
O5	0.006 (2)	0.012 (3)	0.016 (3)	0.000	0.000	0.000

### Geometric parameters (Å, °)

**Table:**

Nb1—O3^i^	1.813 (3)	K1—O3^vii^	2.764 (5)
Nb1—O3^ii^	1.813 (3)	K1—O3^viii^	2.764 (5)
Nb1—O4	2.001 (3)	K1—O3	2.869 (6)
Nb1—O4^iii^	2.001 (3)	K1—O3^ix^	2.869 (6)
Nb1—O1^iv^	2.256 (2)	K1—O2^x^	2.989 (6)
Nb1—O1	2.256 (2)	K2—O5^xi^	2.48 (3)
Nb2—O4^v^	1.849 (3)	K2—O3^xi^	2.630 (9)
Nb2—O4	1.849 (3)	K2—O3^xii^	2.630 (9)
Nb2—O5	1.932 (2)	K2—O2^xiii^	2.70 (2)
Nb2—O3	2.122 (3)	K2—O2^xiv^	2.70 (2)
Nb2—O3^v^	2.122 (3)	Ag1—O5^xi^	2.272 (11)
Nb2—O2	2.128 (4)	Ag1—O2^xiii^	2.500 (10)
As1—O2^i^	1.672 (4)	Ag1—O2^xiv^	2.500 (10)
As1—O2^vi^	1.672 (4)	Ag2—O5^xi^	2.188 (15)
As1—O1	1.707 (4)	Ag2—O2^xiii^	2.386 (13)
As1—O1^v^	1.707 (4)	Ag2—O2^xiv^	2.386 (13)
			
O3^i^—Nb1—O3^ii^	102.60 (18)	O4^v^—Nb2—O3	169.44 (12)
O3^i^—Nb1—O4	95.73 (12)	O4—Nb2—O3	90.79 (12)
O3^ii^—Nb1—O4	93.90 (12)	O5—Nb2—O3	86.27 (14)
O3^i^—Nb1—O4^iii^	93.90 (12)	O4^v^—Nb2—O3^v^	90.79 (12)
O3^ii^—Nb1—O4^iii^	95.73 (12)	O4—Nb2—O3^v^	169.44 (12)
O4—Nb1—O4^iii^	164.57 (17)	O5—Nb2—O3^v^	86.27 (14)
O3^i^—Nb1—O1^iv^	164.38 (12)	O3—Nb2—O3^v^	79.39 (16)
O3^ii^—Nb1—O1^iv^	92.92 (12)	O4^v^—Nb2—O2	91.67 (11)
O4—Nb1—O1^iv^	84.79 (13)	O4—Nb2—O2	91.67 (11)
O4^iii^—Nb1—O1^iv^	82.70 (13)	O5—Nb2—O2	166.61 (19)
O3^i^—Nb1—O1	92.92 (12)	O3—Nb2—O2	83.44 (11)
O3^ii^—Nb1—O1	164.38 (12)	O3^v^—Nb2—O2	83.44 (11)
O4—Nb1—O1	82.70 (13)	O2^i^—As1—O2^vi^	106.2 (3)
O4^iii^—Nb1—O1	84.79 (13)	O2^i^—As1—O1	110.25 (10)
O1^iv^—Nb1—O1	71.64 (15)	O2^vi^—As1—O1	110.25 (10)
O4^v^—Nb2—O4	98.72 (19)	O2^i^—As1—O1^v^	110.25 (10)
O4^v^—Nb2—O5	97.03 (14)	O2^vi^—As1—O1^v^	110.25 (10)
O4—Nb2—O5	97.03 (14)	O1—As1—O1^v^	109.6 (3)

Symmetry codes: (i) −*x*+1/2, *y*+1/2, *z*; (ii) −*x*+1/2, −*y*+1/2, −*z*; (iii) *x*, −*y*+1, −*z*; (iv) −*x*, −*y*+1, −*z*; (v) *x*, *y*, −*z*+1/2; (vi) *x*−1/2, *y*+1/2, *z*; (vii) −*x*, −*y*, −*z*; (viii) *x*, −*y*, −*z*; (ix) −*x*, *y*, *z*; (x) *x*−1/2, −*y*+1/2, −*z*; (xi) −*x*, −*y*, −*z*+1; (xii) *x*, −*y*, −*z*+1; (xiii) −*x*+1/2, −*y*+1/2, −*z*+1; (xiv) *x*−1/2, −*y*+1/2, −*z*+1.
